# Vaccination

**Published:** 1870-11

**Authors:** 


### VACCINATION.

In the observation of six of the most eminent medical men in the world, with an experience of over four hundred thousand vaccinations, there was not a single case where another disease had been conveyed; on the contrary, there is evidence to believe that vaccination hardens the constitution, as small-pox certainly does. And as millions of persons who have been efficiently vaccinated have escaped small-pox, common sense dictates the propriety of vaccinating every child before it is two months old.

